# Notes on Poor-Law Administration

**Published:** 1905-01-14

**Authors:** 


### NOTES ON POOR-LAW ADMINISTRATION.

### THE FOOD ALLOWANCE IN INSTITUTIONS.

Few things cause more trouble than the allowance of food to the inmates and officials of charitable institutions; few things are more difficult to arrange with due regard to reason and economy. Lately the nurses at the Newport (Salop) Infirmary sent in a protest to the Guardians complaining of the limited amount of their food, and stating that they are fed less generously than the inmates. They also submitted the following weekly dietary table which they wish adopted for their use. Bread, 7 lb.; meat, 5¼ lb.; flour, 1 lb.; butter, 1 lb.; lump sugar, 1 lb.; moist sugar, 1 lb.; bacon, 1 lb.; jam, ½ lb.; cheese, ¼ lb.; tea, 6 oz.; coffee, 6 oz., or 3 oz. of cocoa; rice, etc., 6 oz.; eggs, 6; currants and raisins, 2 oz.; fruit and vegetables, at master's discretion; milk, 10 pints; condiments, as required; fish, fowl, or rabbit, in lieu of meat. As no beer was allowed, or anything in lieu thereof, an allowance of 30s. would be appreciated. Some of the items of this table would seem to the ordinary housekeeper unreasonably lavish, such, for example, as the demand for 6 oz. of tea weekly in addition to 6 oz. of coffee or 3 oz. of cocoa, and 1 lb. of moist sugar, the last evidently meant only to be used in puddings, as there is also 1 lb. of loaf. Nor does it seem sensible to specify a weekly allowance of 6 oz. of rice per head and 2 oz. of currants and raisins. Do the nurses want rice-pudding always? Would no other farinaceous pudding be welcome? The general complaint about institutional food is just its monotony, but the Newport nurses seem to be inviting the stereotyping of that monotony. In fact, it is a pity thus to tie the hands of those who cater for their food. It is necessary that the steward should have a list with the maximum quantities allowed per head weekly for each class of inmates and officials, but it is a mistake to make this maximum demand equivalent to a claim of right on the part of each individual, or a reason for the steward and matron allowing the maximum quantities to be given out without inquiring if they are properly used. It is this lack of elasticity which leads both to waste and to theft, as was shown by the scandal at Horton Asylum, and in many cases where the matter has been settled without so much publicity. It seems to us that the most reasonable plan would be to have a cost-limit per head for the various classes of officials, and a general outline of the menu for each meal without absolutely specifying all the items. Thus, it would be taken for granted that the nurses would have bacon, eggs, or fish for breakfast, and the relative cheapness of these and their suitability for the time of year would decide which would appear most frequently. A similar flexibility with regard to other meals would probably give greater comfort than is often found now, and at no greater expense. Certainly this plan would demand more thought from the matron, who would have to let the steward know in plenty of time what she wanted for a given day, and might also give a little more work in the kitchen, where a careful manager would make use of left-over dishes in some new form, adding to the variety of the diet without increasing its cost. The Newport nurses ask for "fish, fowl, or rabbit in lieu of meat," which would seem to indicate that they feel the constant routine of beef and mutton monotonous. In many institutions fish is always served on Friday, and as this rule meets the scruples of some and offends no one, it might reasonably be made general. Poultry is usually regarded as something of a luxury, but with good management might be provided occasionally. As for rabbits, there should be an alternative dish on the table when they are served, as some people dislike them. In short, the whole note of the housekeeping should be as much flexibility as possible. Anæmia and prostration among nurses are often due to insufficient feeding, and this in turn is due not to a lack of supply so often as to a lack of variety, the sameness of the food making it unappetising. Therefore, we think that the Newport nurses have made a mistake in demanding fixed quantities of specified articles, and we hope that the committee to whom the question is referred will give a report which will make it clear to the steward and matron of the infirmary that the nurses are to have good and sufficient food at a reasonable cost, but will neither fix a specific allowance per head, not limit the choice of articles of diet to those named in the table presented.

### FEMALE RELIEVING OFFICERS.

It is customary now in many unions for maternity cases to be interviewed by the lady guardians only, and it is generally felt that this is a desirable change, and one which saves unfortunate girls, who will in any case have enough to pay for their lapse from virtue, from an inquisition which is likely to destroy the remnants of their self-respect. But there are always inquiries as to settlement, putative paternity, and the like, which must be made by an officer of the union, and all these officers are men. Miss Carryer, one of the Leicester guardians, proposed the appointment of a female relieving officer to deal with such cases, and though her motion was lost by the casting vote of the chairman it embodies a further step in the direction of decency, and will probably be heard of again. In addition to these cases, Miss Carryer suggested that the female relieving officer should accompany female lunatics to asylums, and visit the homes of the scrubbers and other female daily workers employed at the infirmary—presumably to see that they were free from communicable disease.

